# Ca^2+^ mediates transcription factor *PuDof2.5* and suppresses stone cell production in pear fruits

**DOI:** 10.3389/fpls.2022.976977

**Published:** 2022-08-24

**Authors:** He Zhang, Siyang Gao, Tianye Wang, Mingyang Xu, Xinyue Li, Guodong Du

**Affiliations:** ^1^Key Laboratory of Fruit Postharvest Biology, Liaoning Province, College of Horticulture, Shenyang Agricultural University, Shenyang, China; ^2^General Station of Agricultural Technology Extension, Xinjiang Production and Construction Corps, Urumqi, China

**Keywords:** pear, Ca^2+^, *PuDof2.5*, *PuPRX42-like*, lignin, stone cell

## Abstract

Stone cells are sclerenchyma cells formed by deposition of lignin, which is the most significant factor limiting the quality of pears. Ca^2+^ was known to inhibit stone cells in pear fruits, but the underlying molecular mechanism remains unclear. Our study revealed that exogenous CaCl_2_ (Ca^2+^) treatment of “Nanguo” pear (*Pyrus ussuriensis*) suppressed the synthesis of lignin and stone cell production. We further analysed the transcriptomes using RNA-seq, identified a transcription factor, *PuDof2.5*, and its targets gene *PuPRX42-like* (lignin polymerase gene) expression decreased in CaCl_2_-treated samples, which are involved in suppressing lignin biosynthesis in pear fruit. *PuDof2.5* was found to bind directly to the *PuPRX42-like* promoter and induced its transcription. Taken together, our results revealed that Ca^2+^ modulated the key lignin biosynthetic transcription factor *PuDof2.5* to suppress stone cell production in pear fruits.

## Introduction

The Nanguo pear (*Pyrus ussuriensis* Maxim) is one of the most popular pears in northern China, and it is deeply loved by consumers for its excellent taste and flavor (Wei et al., 2017). The stone cell content of the Nanguo pear is relatively high, which reduces fruit quality and results in rough flesh texture, lowering its economic value. Therefore, minimizing the stone cell content is essential to improve Nanguo pear quality.

Stone cells are formed from parenchymal cells by deposition of lignin (Rogers and Campbell, 2004). Stone cell production is closely correlated with lignin synthesis, transfer, and deposition (Barros et al., 2015). The biosynthetic pathway of lignin has been studied in pear fruits (Cai et al., 2010). The process starts with the formation of phenylacrylic acid (PA) by the enzyme phenylalanine ammonia lyase (PAL) from L-Phe (L-phenylalanine); cinnamate 4-hydroxylase (C4H) and 4-hydroxycinnamate-CoA ligase (4CL) catalyze the conversion of PA, cinnamyl alcohol dehydrogenase (CAD), and other key enzymes form lignin monomers; finally, the monomers are converted to lignin complexes by peroxidase (PRX) and laccase (LAC).

The key role of PRX genes in plant cell wall lignification has been proved (Shigeto and Tsutsumi, 2016). In Arabidopsis (*Arabidopsis thaliana*), cell-specific downregulation of the peroxidase *AtPRX64* causes a marked delay in the formation of the casparian band, a lignin-based paracellular diffusion barrier in plants (Lee et al., 2019). Overexpression of *FaPRX27* in strawberry fruit, supporting peroxidase involved in the root and fruit lignification (Ring et al., 2013). In the loss-of-function *Arabidopsis* mutant, *PRX17* showed lower lignin content in leafs, while overexpression of *PRX17* showed the opposite phenotype (Cosio et al., 2017). These findings suggested a broader role of *PRXs* genes in lignin biosynthesis.

The DNA-binding One Zinc Finger (Dof) is a family of plant-specific transcription factors that bind to the promoter of their targeted genes at the consensus sequence AAAG (Yanagisawa and Schmidt, 1999). These transcription factors also participate in various developmental processes and respond to environmental stimuli in plants (Khaksar et al., 2019). To date, Dof TF-regulated lignin production has been demonstrated in several plants (Rogers et al., 2005; Li et al., 2019; Cao et al., 2020). In *Arabidopsis*, the double mutants *VDof1* and *VDof2* showed enhanced lignin deposition, suggesting VDof1 and VDof2 as negative regulators of lignin deposition (Ramachandran et al., 2020). However, PuDof2.5, which regulates the stone cell biosynthesis mechanism in Nanguo pear, is not fully understood.

Calcium is a secondary messenger that participates in cell signaling pathways and is closely related to cell wall development, which plays an important role in regulating fruit quality (Wójcik et al., 2014). Modulation of calcium has been reported in terms of the fruit quality of several pear species (Sajid et al., 2019; Zudaire et al., 2019; Dalzochio et al., 2021). Meanwhile, Ca^2+^ also regulates the lignin synthesis process. For example, exogenous application of.5% CaCl_2_ in “Niitaka” pear fruits can decrease PRX enzyme activity to inhibit lignin synthesis (Lee et al., 2007). In “Whangkeumbae” pear, CaCl_2_ significantly reduced PAL, CAD, and PRX enzyme activities, and the expression levels of *PpCAD1* and *PpCAD2* were downregulated (Lu et al., 2015). Ca^2+^ regulates lignin biosynthetic molecular mechanism in “Nanguo” pear remains unclear.

Several studies have indicated that Ca^2+^ can decrease stone cell content. However, most of them only focused on changes in lignin content and related gene expression. In the present study, we characterized the role of *PuDof2.5* in pear fruits. *PuDof2.5* was lowly expressed in fruits after Ca^2+^ treatment. Finally, stone cells were decreased by inhibiting the expression of lignin synthesis gene *PuPRX42-like*. These findings provide a better understanding of the molecular mechanism by which Ca^2+^ inhibits stone cell production in pear fruits.

## Materials and methods

### Plant materials and treatments

Nanguopear (n = 65) trees in the orchard at the Shenyang Agricultural University (Shenyang, China) were used as test materials in this study. The trees were treated with 5 g/L CaCl_2_, which was sprayed on flowers and fruits at full bloom (FB) and 15 days after full bloom (DAFB) twice. Trees were treated with an equal volume of water as the control. Fruit samples were harvested 20, 35, 50, 65, 80, and 110 DAFB in 2021 and 25, 30, 35, 50, 80, and 125 DAFB in 2020. Fresh fruits were harvested for histochemical analysis and stored at −80°C for further analysis. Three replicates were maintained per treatment with 20 fruits per biological replicate.

### Ca^2+^ localization and paraffin section analysis

Localization of free Ca^2+^ in Ca^2+^-treated and control fruits was observed by fluorescence imaging (Wang et al., 2018). The middle flesh was collected with a blade, washed twice with a HEPES buffer, and incubated with Fluo-3/AM at 4°C for 2 h. Then, all the samples were washed with the HEPES buffer at 25°C. Finally, Fluo-3 fluorescence was scanned with a confocal laser scanning microscope (TCS Sp8; Leica, Germany) using a 488-nm laser light to excite the dye and a 525–530-nm long-pass emission filter to read.

Stone cells were observed in paraffin sections by following previous methods (Liu et al., 2021). The pear fruits were fixed with FAA (formaldehyde, acetic acid, and ethanol), followed by safranin and green staining. Finally, the tissue section was mounted with neutral balsam and observed under a light microscope (Nikon, DS-U3).

### Analysis of stone cell, lignin content, and lignin histochemistry

The stone cell and lignin contents of the pear fruits were determined 20, 35, 50, 65, 80, and 110 DAFB. The content of stone cells was determined with a previous method (Xue et al., 2019). The calculation method is as follows: stone cell content (mg/g) = stone cell dry weight (mg)/flesh weight (g). Lignin content was determined with a previous method (Anderson et al., 2015) and calculated as follows: lignin content (mg.g^−1^) = lignin content (mg)/dry weight (g).

Fresh pears were used as the material and were cut and sliced by hand at six developmental stages (20, 35, 50, 65, 80, and 110 DAFB) to confirm the histochemical location of lignin. The sections were placed in 1% phloroglucinol solution and treated with 30% HCl for 5 min. Images were captured with a handheld camera.

### RNA-sequencing

Total RNA was isolated from CaCl_2_-treated and control fruit flesh 35 DAFB (three biological replicates for each treatment), and a library was constructed and sequenced on an Illumina HiSeq 2000 system at LC-Bio Technology Co., Ltd. (Hangzhou, China). We used the fragments per kb per million reads method to calculate differentially expressed genes. Raw RNA-seq data have been deposited on the NCBI Sequence Read Archive (SRA) under accession number PRJNA 797117.

### RNA extraction and gene expression analysis

The cetyltrimethylammonium bromide (CTAB) method was used to extract total RNA from CaCl_2_-treated and untreated fruit flesh (Jaakola et al., 2001), and cDNA synthesis and gene expression analysis were conducted as previously described (Zhang et al., 2019). qRT-PCR was performed on a 7500 Real-time PCR system (Applied Biosystems, Foster City, United States) using SYBR Green Kit (Takara, Tokyo, Japan). The primers used in gene expression analysis are listed in Supplementary Table S1.

### PRX enzyme activity

The PRX activity in CaCl_2_-treated and control fruit flesh was measured as previously described (Alici and Arabaci, 2016). One PRX unit is defined as the amount of enzyme that causes an absorbance change of 0.001 per minute under the test conditions.

### Subcellular localization

The *PuPRX42-like* and *PuDof2.5* coding regions were fused with GFP to form the *Pro35S:GFP-PuPRX42-like* and *Pro35S:GFP-PuDof2.5* constructs. Respectively, the construct was co-infiltrated with the mCherry-labeled membrane marker (PM Marker) and nuclear marker (mCherry marker) into onion bulb samples by *Agrobacterium tumefaciens*-mediated infiltration, the onion bulb samples were cultured in an MS medium for 48 h after infiltration. GFP fluorescence was observed under a confocal microscope (TCS Sp8; Leica, Germany). For green fluorescence observation, the excitation wavelength was 488 nm and the emission wavelength was 520–540 nm. For red fluorescence observation, the excitation wavelength was 561 nm and the emission wavelength was 610–630 nm. *Pro35S:GFP* was used as a control. All the assays were repeated at least 3 times.

### Functional analysis

To overexpress *PuDof2.5* and *PuPRX42-like* in the Nanguo pear fruits, CDS regions were separately cloned into a pRI101 vector to form *Pro35S:pRI101-PuDof2.5* and *Pro35S: pRI101-PuPRX42-like*. These plasmids were transformed into *A. tumefaciens* strain GV3101, and preparation of infiltration buffer and fruit infiltration were performed as previously described (Li et al., 2016). Briefly, the infiltration buffer was taken with a 1-ml sterile syringe and injected into on-tree Nanguo pear fruits 20 DAFB. For each fruit, one side was used for infiltrating target constructs, and the other side for infiltrating empty pRI101 as control. The infiltrated fruits were harvested 6 day after the infiltration, and the fruit flesh around the infiltrated area was sampled for further use.

### GUS analysis

The *PuPRX42-like* promoter sequence (1,011 bp) was cloned upstream of the GUS gene in the pBI101 vector to obtain the reporter construct. Meanwhile, the *PuDof2.5* CDS was introduced into the pRI101 vector to generate the effector construct. The *A. tumefaciens* strain GV1301 harboring the reporter and effector vectors were infiltrated into *N. benthamiana* leaves (Li et al., 2017). Histochemical staining was performed with X-gluc, and GUS activity was determined as previously described (Li et al., 2016). Stained leaves were viewed with an optical microscope, the infiltration was repeated three times (three biological replicates).

### ChIP-PCR analysis

The *PuDof2.5* CDS was cloned into the pRI101-3xflag vector (Yue et al., 2020) and transformed into an *A. tumefaciens* GV1301 strain. The pear fruits were infected as previously described (Li et al., 2020). ChIP assay was performed using the EpiQuik Plant ChIP Kit (56383; Cell Signaling Technology, Danvers, MA, United States) following the manufacturer′s instructions. Chromatin fragmentation was performed using an Uibra Cell VCX 150PB sonicator (Sonics and Materials Inc., Newtown, CT, United States). The specific for the ChIP-PCR assay was prepared as previously method (Li et al., 2020). Each ChIP assay was repeated three times, and enriched DNA fragments in each ChIP sample were used as one biological replicate for qPCR. Six regions of the *PuDof2.5* promoter were analyzed to assess the enrichment; the primers used are listed in Supplementary Table S1.

### Yeast one-hybrid assays

The *PuDof2.5* CDS sequences were inserted into the pGADT7 vector at the *Nde*I and *Eco*RI restriction sites to generate a prey construct. The *PuPRX42-like* promoter was cloned into the pAbAi vector using the *Sac*I and *Sma*I restriction sites to produce the bait constructs. The specific operation for the Y1H assay was performed as previously described (Ji et al., 2021). All the primers used in this study are listed in Supplementary Table S1.

## Results

### Calcium suppresses lignin and stone cell contents in pear fruits

**Figure 1:**
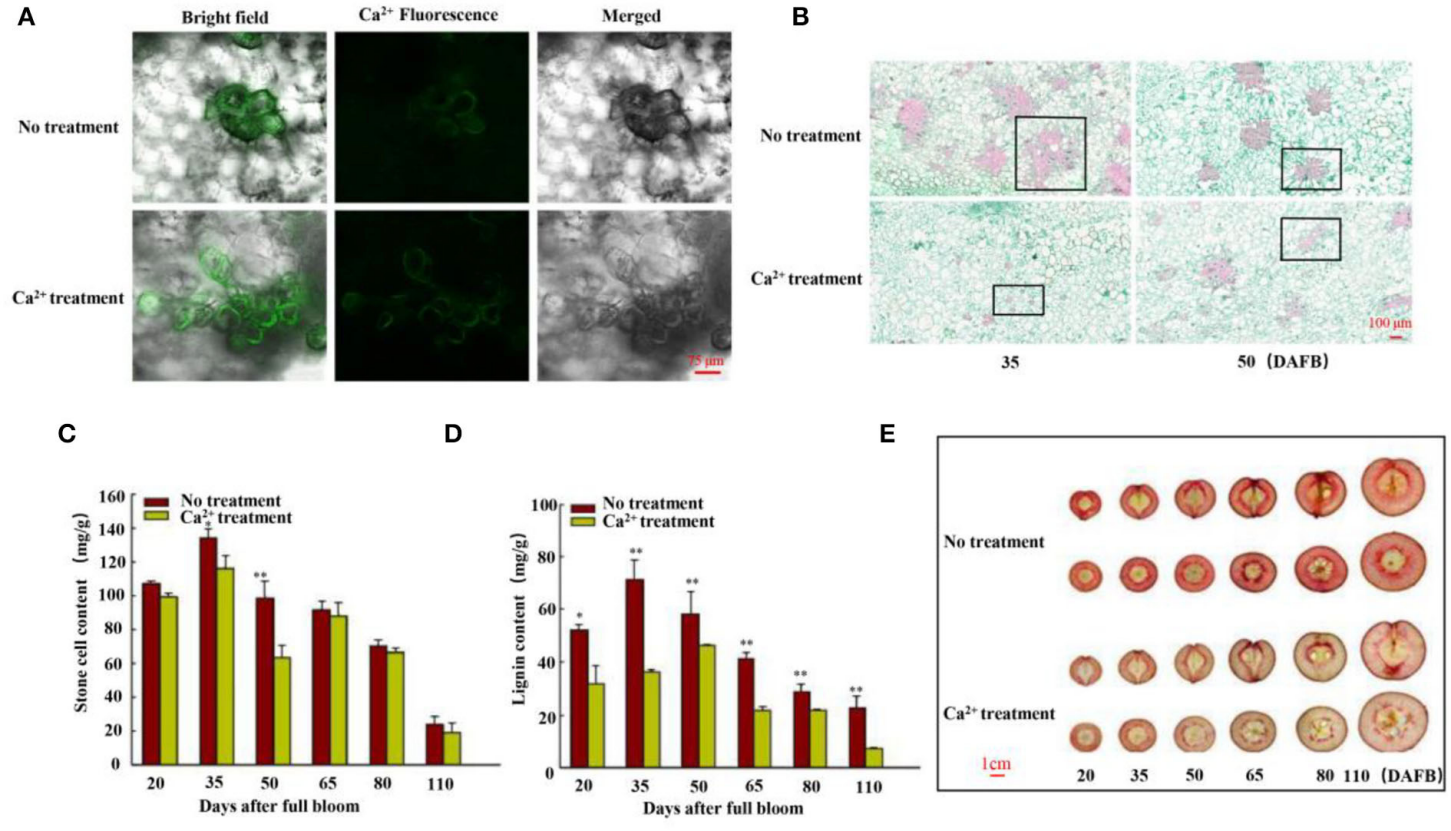
Free Ca^2+^, stone cell morphology, and lignin and stone cell contents. **(A)** Free Ca^2+^ in the flesh cells of fruits was detected using Fluo-3/AM 35 days after full bloom (DAFB). Scale bar 75 μm. **(B)** Morphology of stone cells was observed using paraffin sections of fruits 35 and 50 DAFB. Scale bar 100 μm. **(C)** Stone cell production and **(D)** lignin content of fruits were measured at six stages (20, 35, 50, 65, 80, and 110 DAFB). **(E)** Staining of sections with phloroglucinol-HCl (scale bar 1 cm). Data are shown as means ± SE (n = 3). Statistical significance was determined by Student's *t*-test: ** *P* < 0.01.

The Nanguo fruits on the tree were treated with 5 g·L^−1^ CaCl_2_ in 2020 and 2021. Fruits were harvested 35 days after full bloom (DAFB). The fluo-3/AM staining revealed that flesh cells had higher free Ca^2+^ in CaCl_2_-treated fruits than in control fruits (Figure 1A).

Then, the lignin and stone cell contents of the fruits were measured at six fruit developmental stages, and the findings were consistent with the paraffin section results (Figures 1B–D, Supplementary Figure S2). We further stained the harvested fruits (25, 30, 35, 50, 80, and 125 DAFB in 2020 and 20, 35, 50, 65, 80, and 110 DAFB in 2021) to confirm the results. Hand-cut sections were prepared by phloroglucinol-HCl staining. The lignin-specific histochemical staining indicated that the level of lignification in the control fruits was higher than in the CaCl_2_-treated fruits (Figure 1E, Supplementary Figure S1), indicating fewer stone cell primordia in the CaCl_2_-treated fruits. Thus, the findings collectively indicate that the CaCl_2_ treatment significantly inhibited lignin biosynthesis and stone cell production in Nanguo pear.

### *PuPRX42*-like gene assists calcium in inhibiting lignin biosynthesis

**Figure 2:**
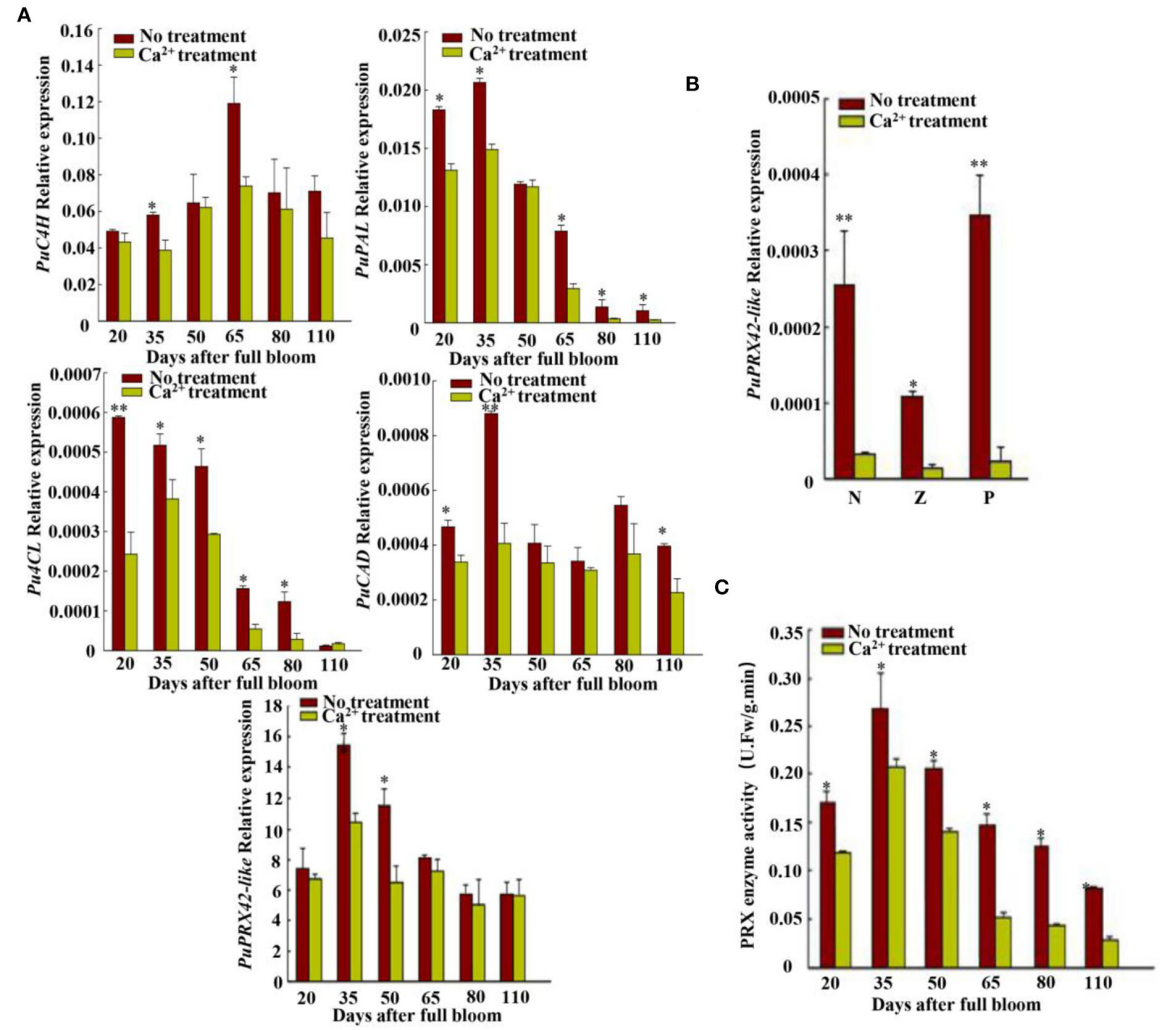
Synthetic lignin relative gene expression and PRX enzyme activity analyses. **(A)** Expressions of lignin biosynthesis genes *PuC4H, PuPAL, Pu4CL, PuCAD*, and *PuPRX42-like* were measured at six fruit developmental stages; *PuC4H*, cinnamate 4-hydroxylase gene; *PuPAL*, phenylalanine ammonia lyase gene; *Pu4CL*, 4-coumarate gene; *PuCAD*, cinnamyl alcohol dehydrogenase gene; *PuPRX*42-like, peroxidase gene. **(B)** *PuPRX42-like* expression in different pear tissues. N, flesh close to the core; Z, flesh in the middle; P, flesh close to the fruit skin. **(C)** PRX enzyme activity at six developmental stages. Data are shown as mean ± SE. Statistical significance was determined by Student's *t*-test: * *P* < 0.05, ** *P* < 0.01.

To explore how CaCl_2_ inhibits lignin production from 20 to 125 DAFB in Nanguo pear fruits, we measured the PAL, C4H, 4CL, CAD, and PRX activities of the CaCl_2_-treated and control fruit samples (Supplementary Figure S3). The CaCl_2_ treatment significantly inhibited PRX activity (Figure 2C), and PRX activity showed the highest correlation with lignin and stone cell contents (Table 1). Subsequently, the expression of lignin biosynthesis genes *PuPRX42-like, PuPAL, PuC4H, Pu4CL*, and *PuCAD* was analyzed by qRT-PCR (Figure 2) to identify the key genes that contribute to the lower lignin accumulation in CaCl_2_-treated fruits. The expression level of *PuPRX42-like* was lower in the CaCl_2_-treated fruits than in the control fruits and consistent with PRX activity (Figures 2A,C).

**Table 1:** Correlation analysis between stone cell, lignin content, and lignin-related enzyme activity.

	**Lignin**	**Stone cell**	**PAL**	**C4H**	**4CL**	**CAD**	**POD**
Lignin	1	0.789**	0.684**	0.626**	0.579**	0.517**	0.910**
Stone cell		1	0.749**	0.716**	0.639**	0.620**	0.856**

PAL, PAL enzyme activity; C4H, C4H enzyme activity; 4CL, 4CL enzyme activity; CAD, CAD enzyme activity; POD, POD enzyme activity. Statistical significance was determined by Student's t-test: ^**^ P < 0.01.

**Figure 3:**
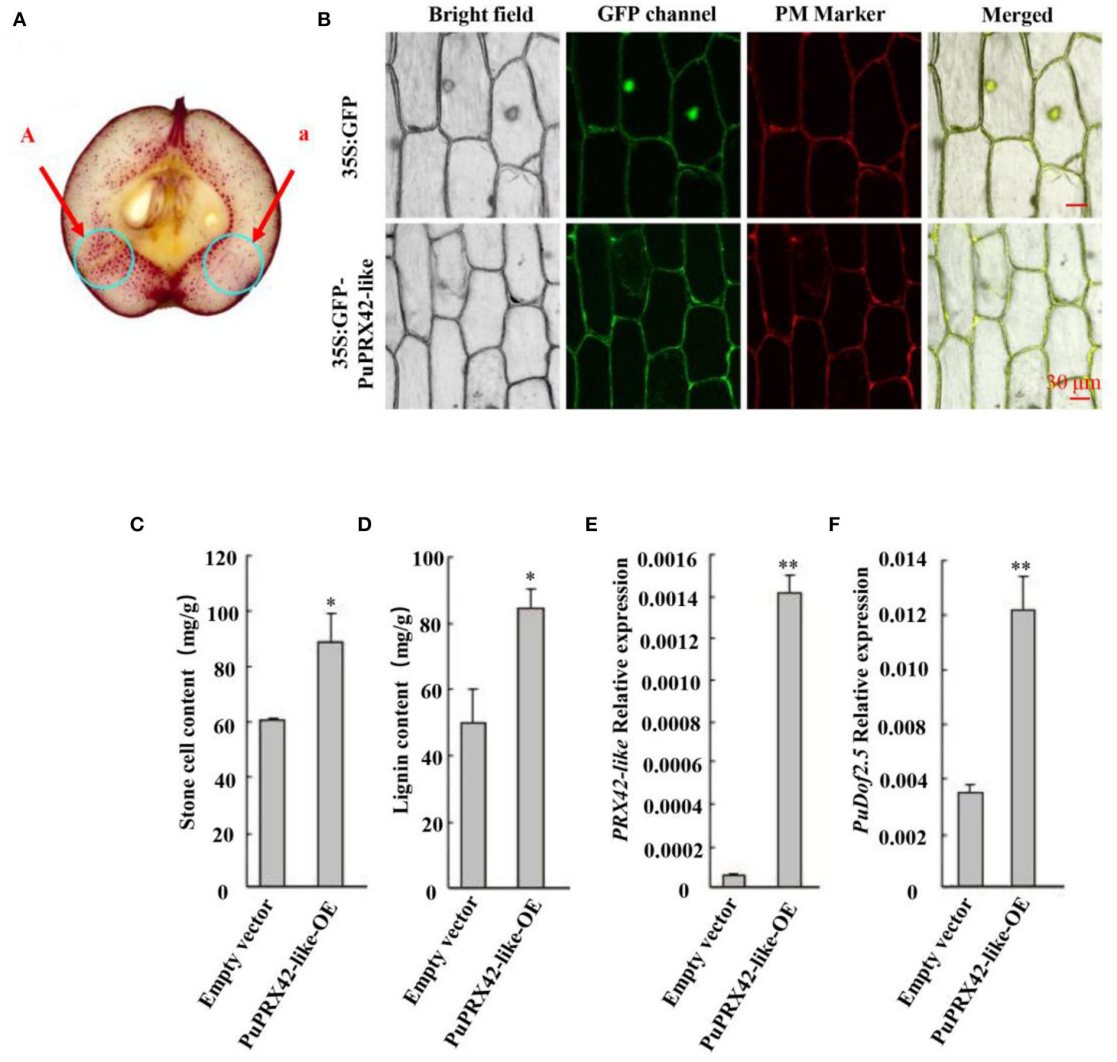
Functional analysis of PuPRX42-like. *PuPRX42-like* was transiently expressed in pear fruits (PuPRX42-like-OE) 30 DAFB following *Agrobacterium*-mediated transient transformation, The transformed pear fruits were harvested 7 days after infiltration to be analyzed by **(A)** staining the sections with phloroglucinol-HCl (A, pRI101-PuPRX42-like; a, pRI101). **(B)** Subcellular localization of *PuPRX42-like* was determined by transiently overexpressing *PuPRX42-like, 35Spro*::GFP, and *35Spro*::*PuPRX42-like*-GFP constructs in onion bulb cells; PM marker served as a membrane marker (scale bar 30 μm). **(C)** Stone cell production and **(D)** lignin content were measured. **(E)** *PuPRX42-like* and **(F)** *PuDof2.5* expression levels in PuPRX42-like-OE fruits were determined by qRT-PCR. Data are shown as mean ± SE (n = 3). Statistical significance was determined by Student's *t*-test: ** *P* < 0.01, * *P* < 0.05.

Furthermore, a transient expression assay was conducted to explore the putative function of *PuPRX42-like* in lignin production. The recombinant plasmid (*35S::PuPRX42-like*-OE) was then transiently transformed into *Agrobacterium* and infiltrated into Nanguo pear fruits on the tree 30 DAFB using the empty vector (pRI101) as the control; The expression of *PuPRX42-like* was significantly higher in *PuPRX42-like*-OE fruits than in the control fruits, consistent with lignin content (Figures 3A,C,D,E). These results indicate that *PuPRX42-like* is important for lignin biosynthesis in pear fruits. *PuPRX42-like* CDS was tagged with GFP and transformed into onion bulb cells following the *Agrobacterium tumefaciens* mediation to further explore its subcellular localization. The analysis of onion bulb cells showed that PuPRX42-like was localized on the membranes (Figure 3B).

### *PuDof2.5* was the key transcription factor after calcium-treated pear fruits

**Figure 4:**
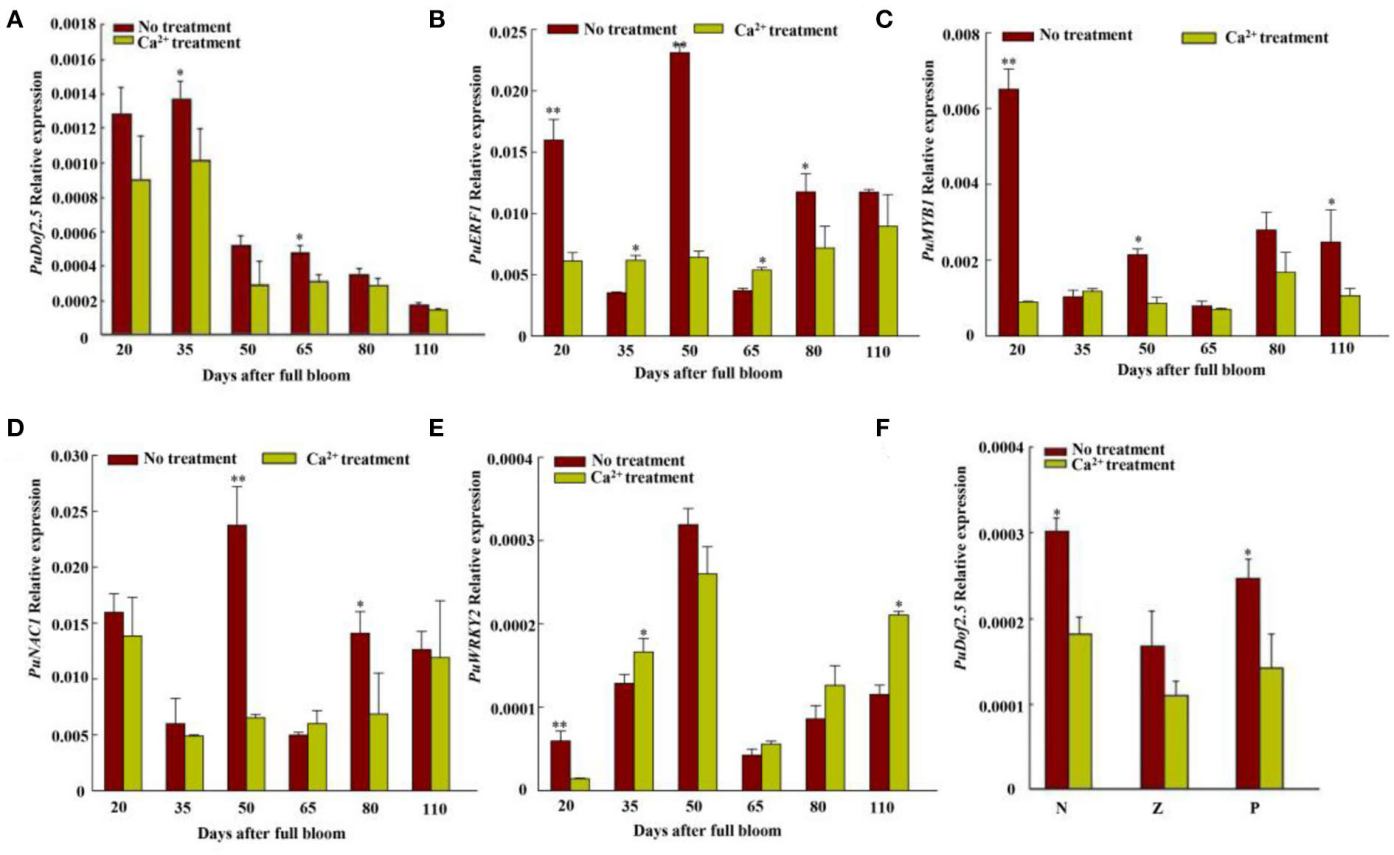
Transcriptome-filtered transcription factor expression level analyses of Nanguo pear fruits. **(A)** Expression level of *PuDof2.5* at six fruit developmental stages after Ca^2+^ treatment. **(B)** Expression level of *PuERF1* at six fruit developmental stages after Ca^2+^ treatment. **(C)** Expression level of *PuMYB1* at six fruit developmental stages after Ca^2+^ treatment. **(D)** Expression level of *PuNAC1* at six fruit developmental stages after Ca^2+^ treatment. **(E)** Expression level of *PuWRKY2* at six fruit developmental stages after Ca^2+^ treatment. **(F)** *PuDof2.5* expression in different pear tissues. N, flesh close to the pit; Z, flesh in the middle; P, flesh close to the fruit skin. Data are shown as mean ± SE (n = 3). Statistical significance was determined by Student's *t*-test: * *P* < 0.05, ** *P* < 0.01.

We compared the transcriptomes of CaCl_2−_treated and control fruits harvested 35 DAFB, and RNA-seq data had different transcription factors, *PuDof2.5, PuWRKY2, PuNAC1, PuERF1*, and *PuMYB1* (Figure 4). Among them, all genes expressions were downregulated after Ca^2+^ treatment, but only *PuDof2.5* expression trend was the same as stone cell change at six fruit developmental stages; these were confirmed by qRT-PCR. We also analyzed *PuDof2.5* expression in different tissues and found a high expression level in flesh close to the core. Furthermore, we identified cis-elements (AAAG) of Dof transcription factor in the promoter of *PuPRX42-like* (1,500 bp) and hypothesized the role of *PuDof2.5* in regulating *PuPRX42-like* expression.

**Figure 5:**
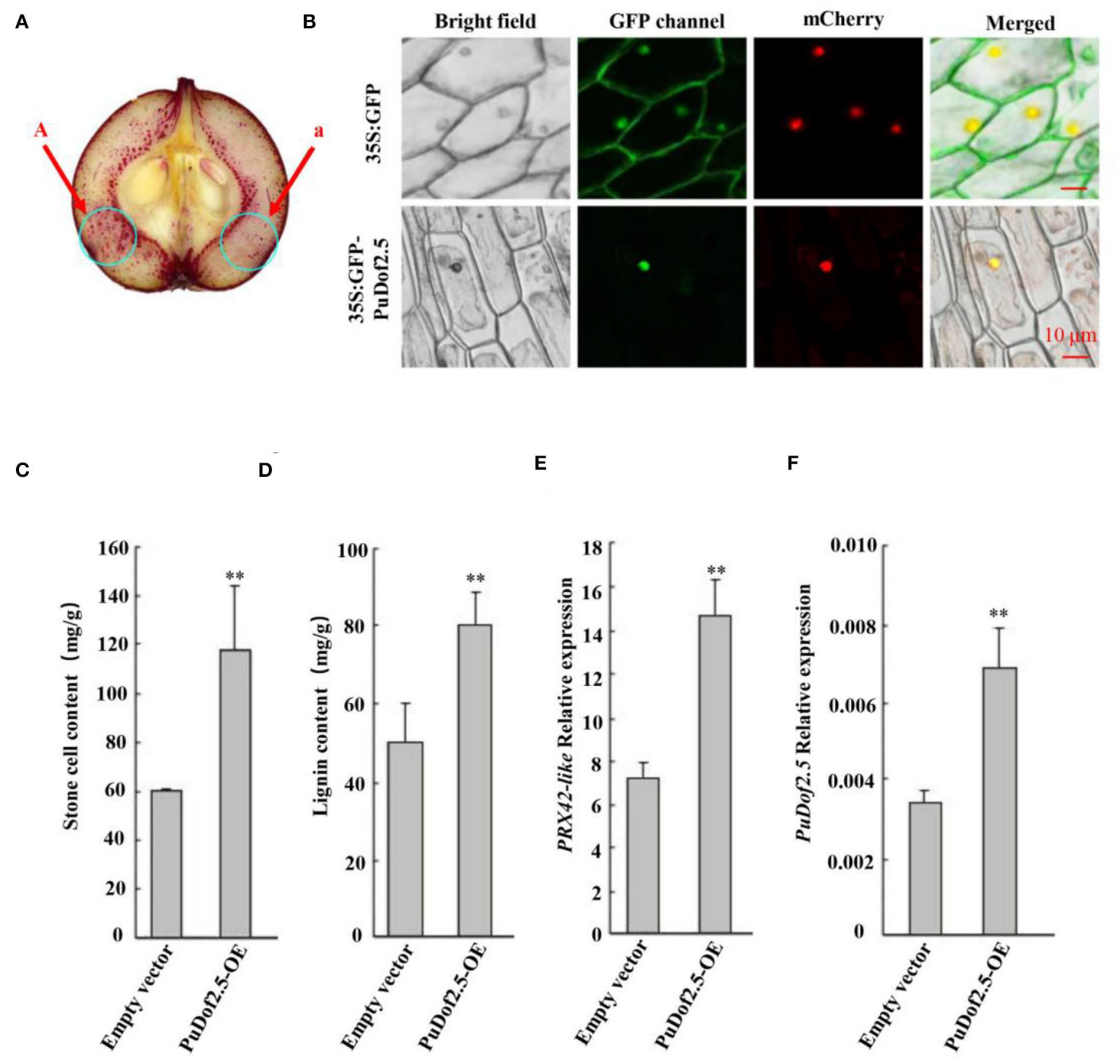
Functional analysis of *PuDof2.5*. **(A)** staining the sections with phloroglucinol-HCl (A, pRI101-PuDof2.5; a, pRI101). **(B)** Subcellular localization of *PuDof2.5* was determined by transiently overexpressing *PuDof2.5, 35Spro*::GFP, and *35Spro*:: *PuDof2.5*-GFP constructs in onion bulb cells; PM marker served as a membrane marker (scale bar 10 μm). **(C)** Stone cell content and **(D)** lignin content were measured. **(E)** *PuPRX42-like* and **(F)** *PuDof2.5* expression levels in PuDof2.5-OE fruits were determined by qRT-PCR. Data are shown as mean ± SE (n = 3). Statistical significance was determined by Student's *t*-test: ** *P* < 0.01.

Furthermore, the *PuDof2.5* CDS was cloned into the pRI101 vector under the control of the 35S promoter to build a 35S: *PuDof2.5* overexpression construct (PuDof2.5-OE). The vector was transformed into *Agrobacterium* and infiltrated into on-tree pear fruits. pRI101 alone was used as control, and qRT-PCR confirmed higher expression of *PuDof2.5* in *PuDof2.5*-OE fruits than in control fruits (Figure 5). We detected that the stone cell and lignin contents in *PuDof2.5*-OE fruits were significantly increased. Notably, the expression level of *PuPRX42-like* was also high in the *PuDof2.5*-OE fruits, suggesting that *PuDof2.5* might play a role in suppressing stone cells by regulating the expression of *PuPRX42-like*.

### *PuDof2.5* binds to the promoter of *PuPRX42*-like

**Figure 6:**
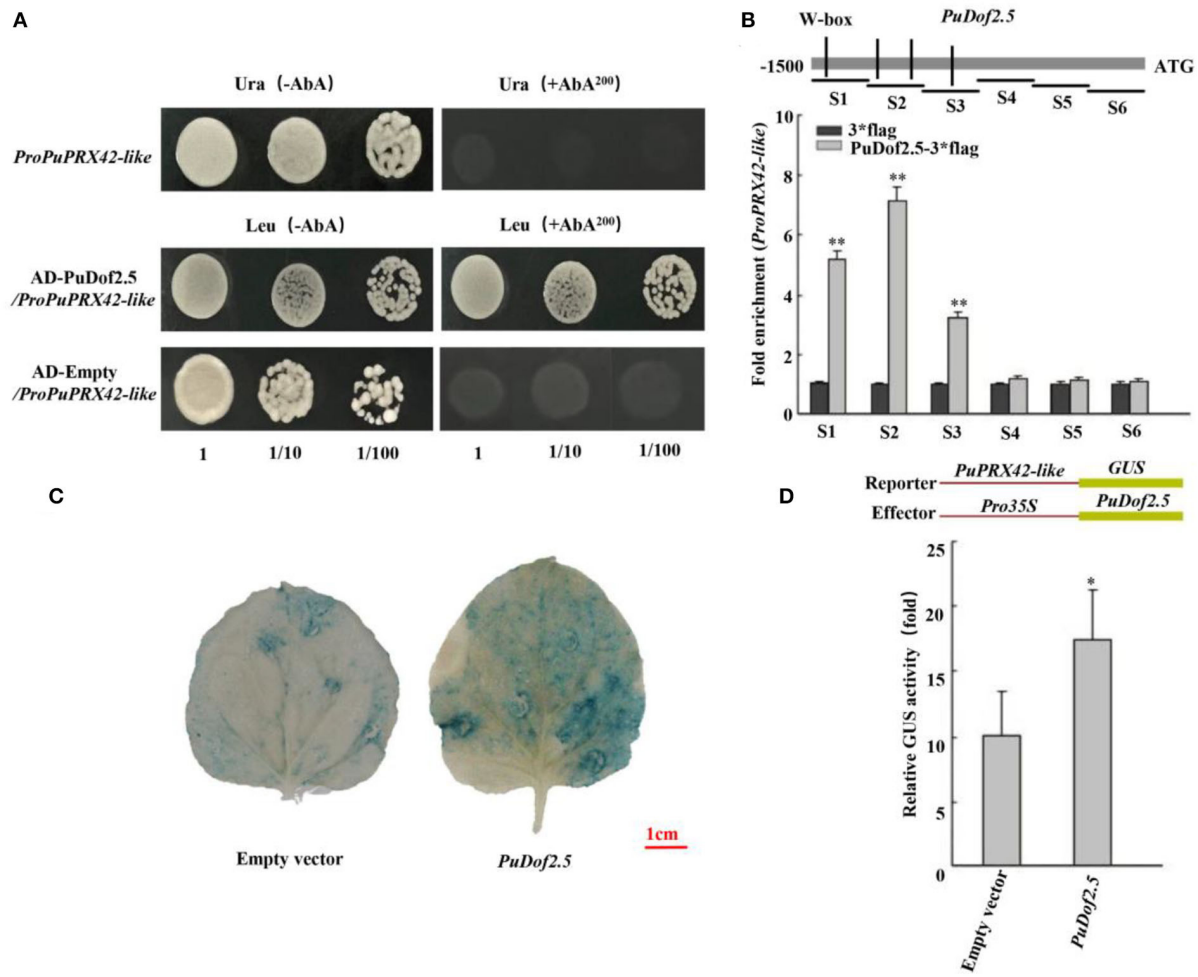
*PuDof2.5* regulates *PuPRX42-like* transcription by promoter binding. **(A)** Y1H analysis of *PuDof2.5* binding to the promoter of *PuPRX42-like*. AbA is a yeast growth inhibitor, and the basal concentration was 200 ng/ml. The empty vector and the *PuPRX42-like* promoter were used as negative controls. **(B)** ChIP-qPCR analysis to determine how *PuDof2.5* directly binds to the *PuPRX42-like* promoter. Eluted DNA was used in the quantitative PCR (qPCR) performed to analyze the six fragments (S1–S6). Immunoprecipitate from cells transiently expressing the vector alone was used as a negative control. **(C)** Histochemical GUS (β-glucosidase) staining. **(D)** GUS activity analysis to determine how *PuDof2.5* promotes the activity of the *PuPRX42-like* promoter. Data are shown as mean ± SE (n = 3). Statistical significance was determined by Student's *t*-test: * *P* < 0.05, ** *P* < 0.01.

Furthermore, a yeast one-hybrid (Y1H) assay was conducted to investigate the binding of *PuDof2.5*. The results showed that *PuDof2.5* bound to the *PuPRX42-like* promoter (Figure 6A). To investigate whether *PuPRX42-like* is a direct target of *PuDof2.5*, ChIP-qPCR assays were performed. The analysis confirmed that *PuDof2.5* could bind to regions of *PuPRX42-like* promoters (Figure 6B). Next, we investigated the *PuDof2.5* regulation to the *PuPRX42-like* promoter by β-glucuronidase (*GUS*) transactivation assay. There was significantly increased activity of the *PuPRX42-like* promoter compared to the empty vector (Figures 6C,D), suggesting that *PuDof2.5* is a transcriptional activator of *PuPRX42-like*. Taken together, the results suggested that *PuDof2.5* directly binds to the *PuPRX42-like* promoter to activate its expression.

## Discussion

Stone cells are a restrictive factor of Nanguo pear fruit quality (Yan et al., 2014). The formation of stone cells is closely related to the deposition of lignin in pear fruits (Li et al., 2007). However, although previous studies have described that Ca^2+^ induces the biosynthesis of lignin in fruit species. CaCl_2_ significantly inhibited lignin biosynthesis enzyme and PAL activities to decrease lignin content in apple fruits (Zhao and Wang, 2015). The studies only investigated changes in stone cell production after exogenous Ca^2+^ treatment and the expression profile of genes involved in lignin biosynthesis and signal transduction. The detailed mechanism by which Ca^2+^ regulates lignin biosynthesis to suppress stone cell production in pear fruits was unclear. Here, we determined that Ca^2+^-inhibited PuDof2.5 induces the expression of a lignin biosynthetic gene (*PuPRX42-like*) by directly binding to its promoter. Our findings suggest that Ca^2+^ has an essential role in reducing stone cell accumulation in pear fruits.

### Calcium reduces stone cell production by reducing lignin accumulation

The texture of pear fruits depends on stone cell mass density and content (Wang et al., 2021). In this study, stone cells developed as clusters, presenting a radial shape throughout the fruit flesh in control fruits, which is consistent with earlier reports (Li et al., 2017). However, Ca^2+^ significantly suppressed stone cell mass content and density (Figure 1B). Higher free Ca^2+^ levels were observed in the Ca^2+^-treated fruits, whereas the levels were lower in the control pear fruits (Figure 1A). The phloroglucinol staining revealed lignin deposition in the flesh of pear fruits and lignified stone cells; the detailed analysis revealed that lignin accumulated rapidly from 20 to 50 DAFB (Figure 1D). Meanwhile, the control fruits were stained darker than the Ca^2+^-treated fruits (Figure 1E), consistent with stone cell content (Figure 1C). A similar result was reported in “Whangkeumbae” (*Pyrus pyrifolia*) pear (Lu et al., 2015). Based on these findings, we proposed that Ca^2+^ regulates lignin accumulation by inhibiting the expression of lignin biosynthesis genes. Together, the studies provided detailed information that Ca^2+^ decreased stone cell content by inhibiting lignin accumulation in pear fruits.

### *PuPRX42*-like is important for Ca^2+^-induced lignin biosynthesis

Studies have proven the role of various enzymes in lignin synthesis. Among PAL, C4H, CAD, 4CL and PRX (Tao et al., 2009), PRX is the key enzyme to catalyze monolignol polymerization into lignin (Dean and Eriksson, 1994; Cheng et al., 2017). The present study analyzed the activities of PRX, PAL, C4H, CAD, and 4CL related to lignin biosynthesis after Ca^2+^ treatment (Supplementary Figure S3). Interestingly, the Ca^2+^ treatment only reduced PRX enzyme activity, and it is consistent with lignin and stone cell contents (Figures 1C,D, 2C, Supplementary Figure S3). An earlier finding with the same results in “Niitaka” pear fruit (Lee et al., 2007) suggested that the PRX enzyme acts a key factor in lignin accumulation to form stone cell. The correlation analysis between lignin synthesis enzyme (PRX, PAL, C4H, CAD, and 4CL) with lignin and stone cell contents also supported this observation (Table 1).

Previous studies have revealed that *PuPRXs* regulated PRX activity and lignin biosynthesis in pear, and that the high expression level of *PuPRX2* promotes lignin accumulation in *PuRBOHF*-overexpressing pear calli (Wang et al., 2021). In our present study, *PuPRX42-like* expression levels were consistent with PRX activity changes (Figures 2A,C). Meanwhile, the overexpression of *PuPRX42-like* significantly accelerated lignin accumulation and stone cell production. PuPRX42-like was localized on the plasma membrane, including sites where lignification occurred. To sum up, the results indicated that *PuPRX42-like* was a key point in the regulation of lignin biosynthesis.

### *PuDof2.5* binds to *PuPRX42*-like promoter and regulates lignin biosynthesis

Dofs are plant-specific transcription factors that act as either transcriptional activators or repressors to influence plant developmental processes (Licausi et al., 2013), i.e., *OsDof15* promoted rice root elongation response to salt stress, and overexpression of *GhDof1* resulted in significant cold tolerance (Su et al., 2017). Meanwhile, overexpression of *AtDof2.1* promoted rice leaf senescence (Zhou et al., 2021). Nonetheless, the role of Dof in regulating lignin biosynthesis in pear fruits is not well-known. We observed that Ca^2+^ significantly suppressed the expression of *PuDof2.5* (Figure 4A). Moreover, *PuDof2.5* is a transcriptional activator that binds to the lignin biosynthetic gene *PuPRX42-like* promoter, directly upregulating its expression (Figures 5A,B,D). Overexpression of *PuDof2.5* increased lignin and stone cell contents and upregulated the expression level of *PuPRX42-like*. Thus, our findings propose that *PuDof2.5* acted as a useful marker to respond to Ca^2+^ treatment.

**Figure 7:**
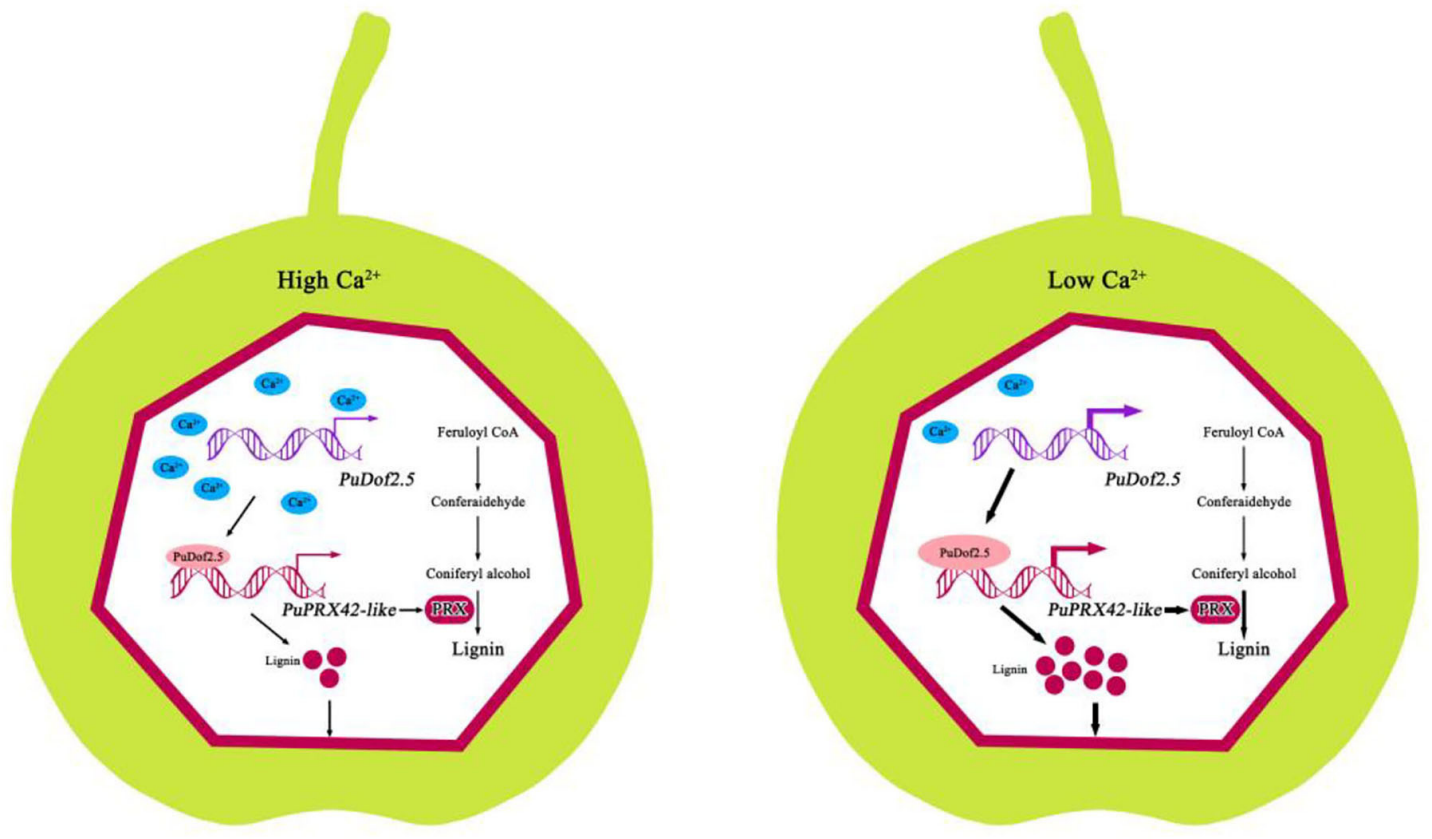
Model of the mechanism by which Ca^2+^ suppresses stone cell production in pear fruits. At high levels of endogenous Ca^2+^, *PuDof2.5* downregulated the transcription of lignin biosynthetic gene *PuPRX42-like* by directly binding to the promoter, decreasing lignin accumulation and stone cell production. At low levels of endogenous Ca^2+^, *PuDof2.5* upregulated the transcription of lignin biosynthetic s *PuPRX42-like*, increasing lignin accumulation and stone cell production.

Our results indicated that Ca^2+^ induced stone production in pear fruits (Figure 7). When endogenous Ca^2+^ levels are high, *PuDof2.5* expression declines, and *PuDof2.5* suppresses the transcription of lignin biosynthetic gene *PuPRX42-like* by direct promoter binding; thus, PRX enzyme activity is reduced, lignin biosynthesis is inhibited, and stone cell production is decreased. When endogenous Ca^2+^ levels decline, *PuDof2.5* expression is enhanced, which accelerates PRX enzyme activity and the transcription of *PuPRX42-like*, leading to a burst of stone cells. Taken together, the results provide a novel insight into the mechanism by which Ca^2+^ regulates stone cell accumulation to improve pear quality.

## Data availability statement

The datasets presented in this study can be found in online repositories. The names of the repository/repositories and accession number(s) can be found in the article/Supplementary material.

## Author contributions

XL and GD designed this study. HZ, TW, SG, and MX performed the experiments. XL, GD, and HZ wrote the manuscript. All the authors read and approved the final version of the manuscript.

## Funding

This study was supported by the National Pear Industry Technology System Project (CARS-28-15), Liaoning Provincial National Natural Science Foundation of China (2019-ZD-0716), Education Department of Liaoning Province Serves Local Projects (LSNFW202009), and Inter-university Cooperation Project of Education Department of Liaoning Province.

## Conflict of interest

Author TW was employed by Xinjiang Production and Construction Corps. The remaining authors declare that the research was conducted in the absence of any commercial or financial relationships that could be construed as a potential conflict of interest.

## Publisher's note

All claims expressed in this article are solely those of the authors and do not necessarily represent those of their affiliated organizations, or those of the publisher, the editors and the reviewers. Any product that may be evaluated in this article, or claim that may be made by its manufacturer, is not guaranteed or endorsed by the publisher.
