# Supplementary material for: Ca2+ mediates transcription factor PuDof2.5 and suppresses stone cell production in pear fruits
Source: Front Plant Sci. 2022 Aug 24;13:976977. doi: 10.3389/fpls.2022.976977 (PMC9449536; doi:10.3389/fpls.2022.976977)
Supplement: Supplementary file 1 [file Data_Sheet_1.docx]

**Supporting Information**

**Figure S1**


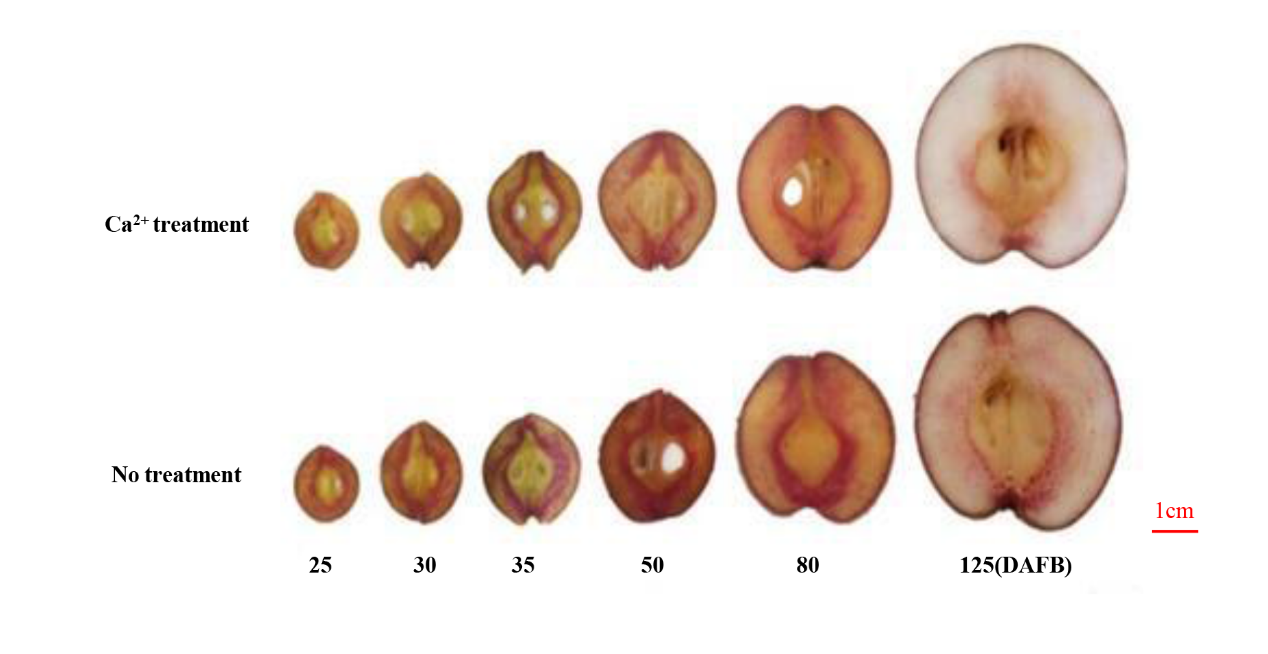


**Fig. S1** The sections were stained by phloroglucinol-HCl at six developmental stages. The fruit were collected at 20, 30, 35, 50, 80, 125 days after full bloom (DAFB) in 2020. Scale bar, 1 cm.

**Figure S2**


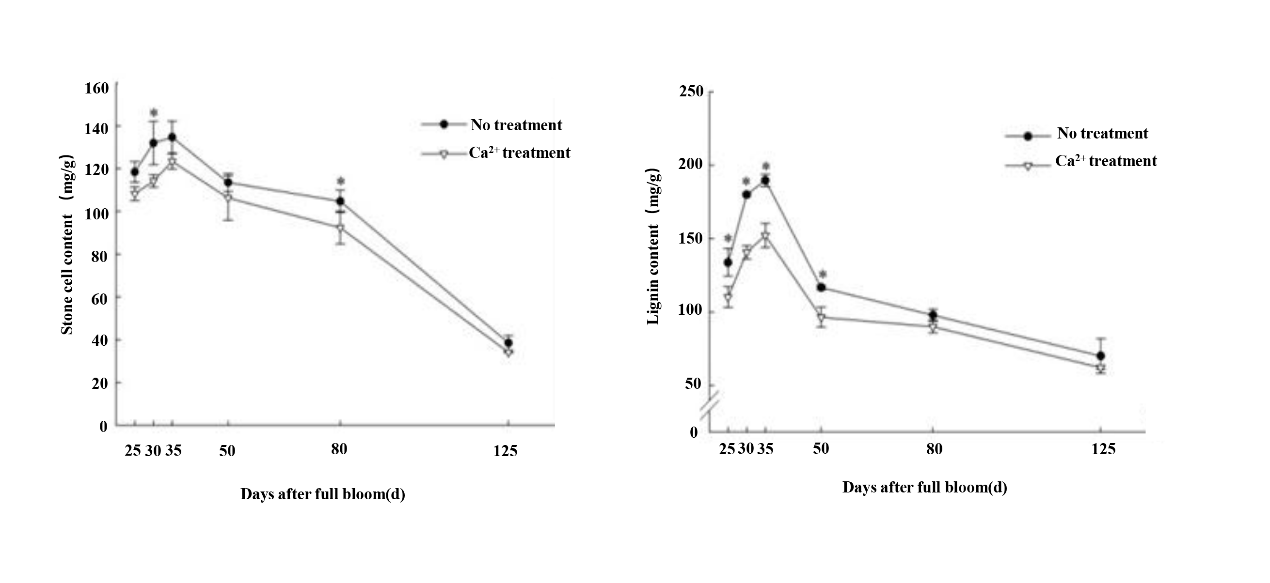


**Fig. S2** Stone cell production and lignin content were measured. The no treatment and Ca^2+^ treatment fruits were collected at 20, 30, 35, 50, 80, 125 DAFB in 2020. Data are shown as mean ± SE. Statistical significance was determined using Student’s *t*-test: *, *P*<0.05.

**Figure S3**


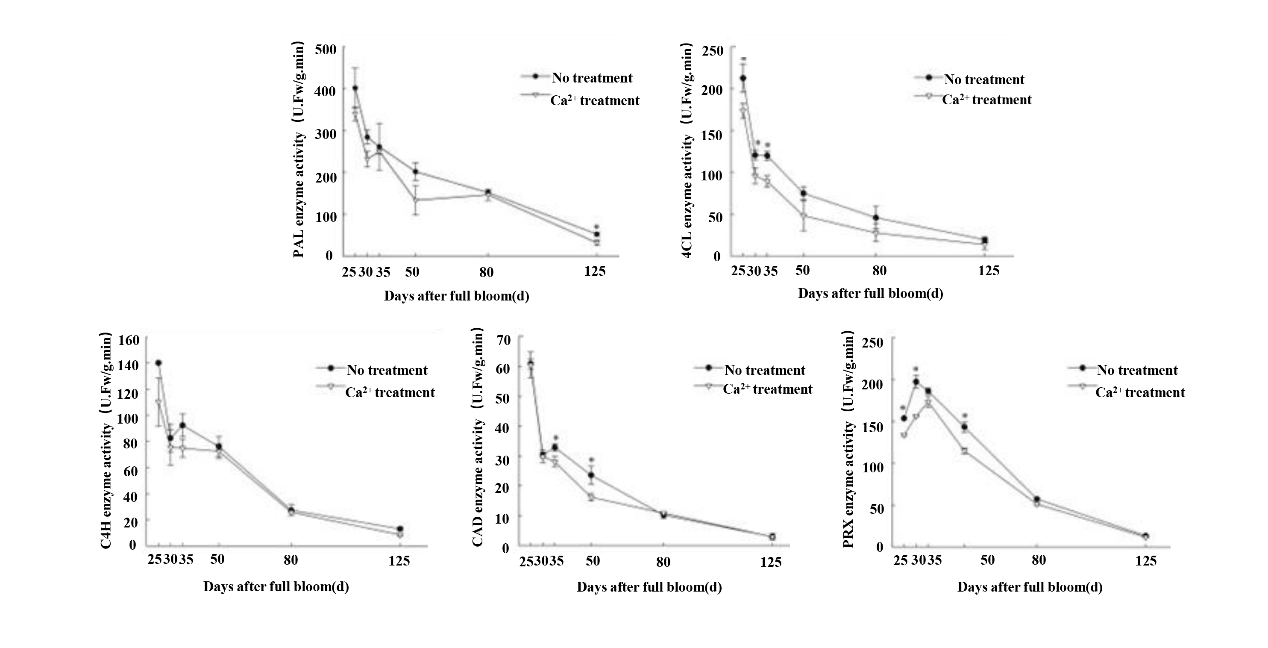


**Fig. S3** Lignin biosynthesis related enzyme PAL (phenylalanine ammonialyase), 4CL (4-hydroxycinnamate-CoA ligase), C4H (cinnamate 4-hydroxylase), CAD (cinnamyl alcohol dehydrogenase), PRX (peroxidase) activity were measured. Data are shown as mean ± SE. Statistical significance was determined using Student’s *t*-test: *, *P*<0.05.

**Figure S4**


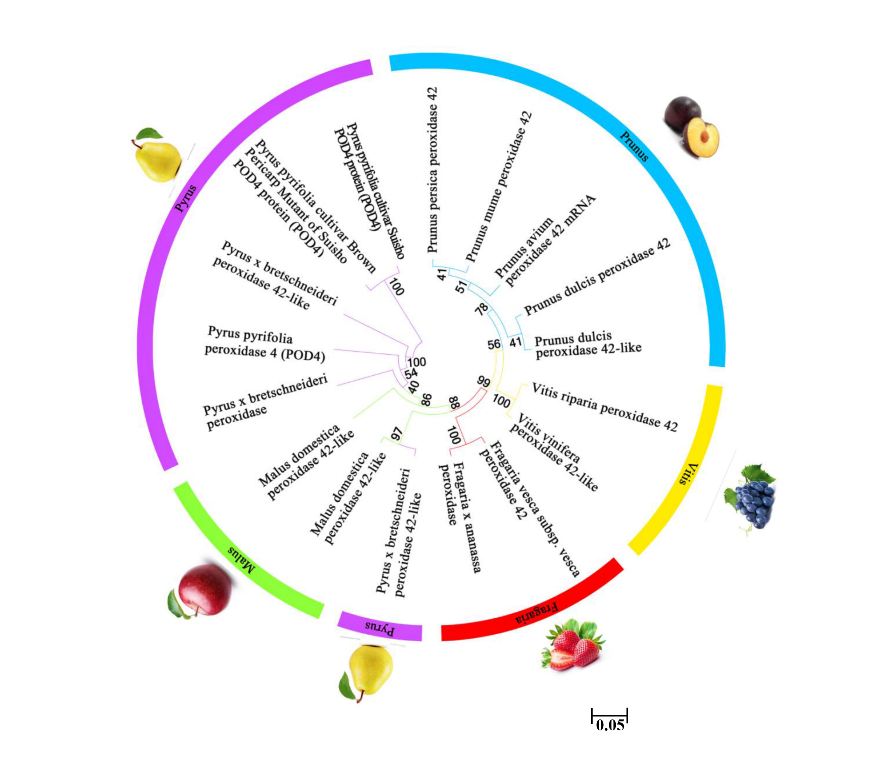


**Fig. S4** Phylogenetic tree of *Pu**PRX42-like* sequences of different fruit crops, based on a conservative approximate alignment. The scale indicates the genetic distance.

**Figure S5**


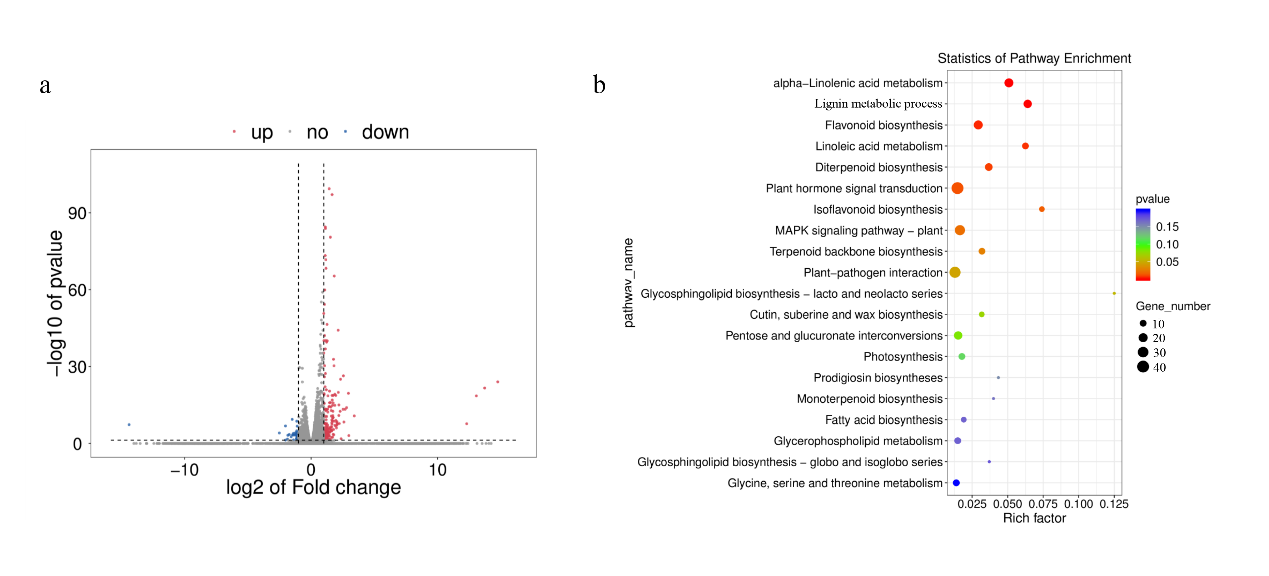


**Fig. S5** RNA-seq data analysis of Ca^2+^-treated and control pear fruits of days after full bloom 35 d. (a) Volcano plot of differently expressed genes, red points represent up-regulated DEGs, The y-axis and x-axis present the -log10(pvalue) and the log2(fold change), blue points represent down-regulated DEGs, and gray points represent non-DEGs. (b) Enriched KEGG pathways among the DEGs. The y-axis and x-axis present the KEGG pathways and the rich factors, respectively. Dot size corresponds to the number of distinct genes, whereas dot color reflects the p-value.

**Table S1** Details of primers used for this study.

| Primer name | Sequence (5’-3’) | Purpose |
| --- | --- | --- |
| Dof2.5-AD-*EcoR*Ⅰ-F | GTACCAGATTACGCTCATATGATGGATACTGCTCAATGGCTGC | Y1H assay |
| Dof2.5-AD-*Nde*Ⅰ-R | ATGCCCACCCGGGTGGAATTCTCACCACGAGCCTCCACCT |  |
| PRX42-like-pABAi-*Sma*Ⅰ-F | CTTGAATTCGAGCTCGGTACCTTGATTTAGGTATCACGCAATTT |  |
| PRX42-like-pABAi-*Sac*Ⅰ-R | AGCACATGCCTCGAGGTCGACGGAAGAAGAAGAAGAAGCCATGGC |  |
| Dof2.5-pRi-*Sac*Ⅰ-F | TCTTCACTGTTGATACATATGATGGATACTGCTCAATGGCTGC | Transient in fruit |
| Dof2.5-pRi-*Nde*Ⅰ -R | CGATCGGGGAAATTCGAGCTCTCACCACGAGCCTCCACCT |  |
| PRX42-like-pRi-*Sac*Ⅰ-F | TCTTCACTGTTGATAATGGCTTCTTCTTCTTCTTCCAGA |  |
| PRX42-like-pRi-*Nde*Ⅰ-R | CGATCGGGGAAATTCCTAGTCCCGGATTTTATTGGCA |  |
| Dof2.5-pRi-*Sac*Ⅰ-F | TCTTCACTGTTGATACATATGATGGATACTGCTCAATGGCTGC | GUS transactivation assay |
| Dof2.5-pRi-*Nde*Ⅰ-R | CGATCGGGGAAATTCGAGCTCTCACCACGAGCCTCCACCT |  |
| PRX42-like-pbi-SalⅠ-F | GACCATGATTACGCCAAGCTTTTGATT TAGGTATCACGCAATTT |  |
| PRX42-like-pbi-BamHⅠ-R | ACTGACCACCCGGGGATCCGGAAG AAGAAGAAGAAGCCATGGC |  |
| ChIP-PRX42-like-S1-F | GATAATTTCAAATAGCGATATTCAGTTTT | ChIP PCR assay |
| ChIP-PRX42-like-S1-R | TTGATTTAGGTATCACGCAATTTTATT |  |
| ChIP-PRX42-like-S2-F | TGATGATTGCATGCCTCCTCA |  |
| ChIP-PRX42-like-S2-R | TGAAATTATCGTTTCCTCTAATAGTTTCT |  |
| ChIP-PRX42-like-S3-F | CAACACTCATTTTTTACACGTGTTGC |  |
| ChIP-PRX42-like-S3-R | CAATCATCATAGTTTTTGTGATGATGC |  |
| ChIP-PRX42-like-S4-F | TACAAGTATTCAGTATATTTTCTTAAAGTTTCTT |  |
| ChIP-PRX42-like-S4-R | ATGAGTGTTGCAGAAATGAGAATGT |  |
| ChIP-PRX42-like-S5-F | AGCATTCACCGCTCTAATTAAACTAC |  |
| ChIP-PRX42-like-S5-R | TACTTGTAGCTAACAGAAAACTTAATGCA |  |
| ChIP-PRX42-like-S6-F | GGAAGAAGAAGAAGAAGCCATGG |  |
| ChIP-PRX42-like-S6-R | GTGAATGCTGCGGTGAATCC |  |
| PRX42-like-full-F | ATGGCTTCTTCTTCTTCTTCC | Gene cloning |
| PRX42-like-full-R | CTAGTCCCGGATTTTATTGG |  |
| Dof2.5-full-F | ATGGATACTGCTCAATGGCTGC |  |
| Dof2.5-full-R | TCACCACGAGCCTCCACCT |  |
| PRX42-like-ex-F | TCTGTCAATCTGCTGCTGT | Gene expression |
| PRX42-like-ex-R | TCATTCAAATCAGCGTCT |  |
| CAD-ex-F | AAGGAAACTGAGGAGATGCTTGAAT |  |
| CAD-ex-R | TACTTTATTAAATAAGATTGCTGCCG |  |
| C4H-ex-F | GCACAGCAGAAGGGAGAAATC |  |
| C4H-ex-R | GTCGGGCTCTGTGATTTGTAC |  |
| PAL-ex-F | GAAGTGCTACAGAATCAG |  |
| PAL-ex-R | GAATCTTATGCCAGAGTAG |  |
| 4CL-ex-F | CATGCTCCTCCTCCAAAACAG |  |
| 4CL-ex-R | TCCTTCACCTTGTCCACGTAA |  |
| NAC1-ex-F | ACACAGACATACAGCGAC |  |
| NAC1-ex-R | TCGCAACCATGGCCATTG |  |
| MYB1-ex-F | CTGGAACTCCACGTTGAAGC |  |
| MYB1-ex-R | AACCGAACTCGTGCTCATCC |  |
| Dof2.5-ex-F | CGACGGTAGCAGGCACTTA |  |
| Dof2.5-ex-R | CTCTACTTCTTCAGGTCCA |  |
| WRKY2-ex-F | CACTCGAGCGTTCCAGAA |  |
| WRKY2-ex-R | ACAGTCGCAGTCCTCTTG |  |
| ERF1-ex-F | ACCCTTGCTATGTGACCC |  |
| ERF1-ex-R | GTTATCAGCTTCCCTCGC |  |
| Actin-F | GCTGGATTTGCTGGTGAT |  |
| Actin-R | GCTCACTATGCCGTGCTC |  |
